# TLR-activated plasmacytoid dendritic cells inhibit breast cancer cell growth *in vitro* and *in vivo*

**DOI:** 10.18632/oncotarget.14315

**Published:** 2016-12-28

**Authors:** Jing Wu, Shuang Li, Yang Yang, Shan Zhu, Mingyou Zhang, Yuan Qiao, Yong-Jun Liu, Jingtao Chen

**Affiliations:** ^1^ Institute of Translational Medicine, The First Hospital, Jilin University, Changchun, 130061, China; ^2^ Department of Cardiovascular Center, The First Hospital, Jilin University, Changchun, 130031, China; ^3^ Sanofi Research and Development, Cambridge, MA, 02139, USA

**Keywords:** breast cancer, plasmacytoid dendritic cells, Toll-like receptor, Imiquimod, CpG

## Abstract

Plasmacytoid dendritic cells (pDCs) are a unique subset of naturally occurring dendritic cells, which triggers the production of large amounts of type I interferons (IFNs) after viral infections through Toll-like receptor (TLR) 7 and TLR9. Recent studies have demonstrated that the activation of pDCs kills melanoma cells. However, the role of activated pDCs in breast cancer remains to be determined. In the present study, we generated mouse models of breast cancer and demonstrated that activated pDCs can directly kill breast tumor cells through TRAIL and Granzyme B. Furthermore, we established that pDCs initiate the sequential activation of NK cells and CD8^+^ T cells, and ultimately inhibit breast tumor growth. Understanding the role of activated pDCs in breast cancer may help to develop new strategies for manipulating the function of pDCs and induce anti-tumor immunity in breast cancer.

## INTRODUCTION

Immunosurveillance can protect humans from tumor development. However, tumors sometimes progress and escape through the immunosurveillance processes [1]. Understanding the paradoxical role of the immune system during cancer development is a precondition for developing new strategies for cancer immunotherapy.

Dendritic cells (DCs) are antigen-presenting cells that play a critical role in linking innate and adaptive immune response. DCs are generally divided into myeloid dendritic cells (mDCs) and plasmacytoid dendritic cells (pDCs) [2]. mDCs are key regulators for maintaining the balance of immunity and tolerance due to their ability to prime B and T lymphocytes, the effector cells of the immune response [3, 4]. pDCs play a key role at the interface of innate and acquired immunity in anti-viral responses by sensing viral infection through Toll-Like Receptors (TLRs) TLR7 and TLR9, which results in the production of large amounts of type-I interferons (IFNs) [5]. PDCs can trigger the activation and differentiation of natural killer (NK) cells, mDCs, B cells and T cells. In addition, pDCs have been shown to mediate tolerance to airway antigens, oral antigens, and cardiac allografts, which is consistent with their antigen presentation capabilities [6, 7].

Functional alterations of pDCs in the tumor microenvironment have been described as a mechanism to escape immunosurveillance [8–10]. Researchers have identified that pDCs in the tumor microenvironment are mainly in a non-activated state, and have been associated with the development and maintenance of an immunosuppressive tumor microenvironment [8, 10–13]. However, some recent studies have implicated pDCs in tumor regression [14–17].

It has been shown that the activation of TLR9 by CpG activated pDCs, which were capable of initiating effective anti-tumor immunity through the activation of NK cells, mDCs and CD8^+^ T cells in a mouse melanoma model [14, 17–19]. TLR7 ligand Imiquimod (IMQ) also activated pDCs that were able to directly eliminate tumor cells in a mouse melanoma tumor model, as well as in melanoma patients [15, 16, 20].

It has been reported that pDCs contribute to the suppressive tumor microenvironment via generation of regulatory T cells and represent an independent prognostic factor associated with poor outcome [10, 21–24]. In NEU15 cell line HER2^+^/neu mouse model, the immunosuppressive activity of pDCs was reverted by intratumoral injection of TLR7 ligand, inducing tumor regression [25]. Currently, the use of TLR7 agonists for clinical purposes is very limited. In the present study, we generated a mouse breast cancer model using TUBO, a HER2/Neu positive breast cancer cell line from BALB/c mice [26, 27]. PDCs were activated by IMQ and CpG *in vitro* to investigate their anti-tumor effect. Our research demonstrates that activated pDCs can kill breast cancer cells *in vitro* through TNF-related apoptosis-inducing ligand (TRAIL) and Granzyme B. In our experiments IMQ was more effective than CpG. We also show that pDCs activate NK cells and CD8^+^ T cells, and ultimately inhibit the growth of breast cancer cells. Understanding the function of activated pDCs in anti-tumor immunity may help to develop new strategies for manipulating the function of pDCs and inducing anti-tumor immunity in breast cancer.

## RESULTS

### Morphological, phenotypic and functional changes of pDCs after activation by IMQ and CpG

**Figure 1:**
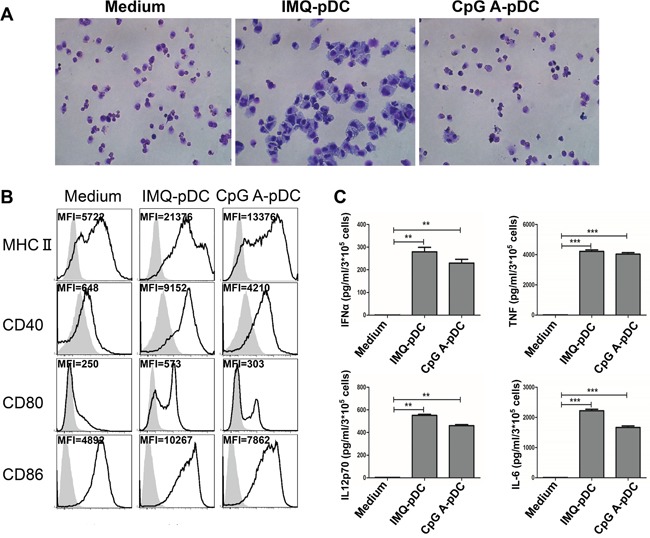
Morphologic, phenotypic and functional changes of pDCs after activation with IMQ and CpG pDCs were harvested after activation with IMQ and CpG for 48 hours, and assessed for morphologic changes by Giemsa staining **A**. and for phenotypic changes by flow cytometry **B**. **C**. Supernatant from pDCs culture medium were collected after activation with IMQ and CpG for 36 hours to detect the release of IFN-α by ELISA and IL-12p70, TNF-α, and IL-6 by CBA. Data shown are expressed as mean ± SEM, and represent three independent experiments with similar results. Paired t-test was used for statistical comparison, ^*^P<0.01, ^**^*P<0.001.

In order to determine the morphologic, phenotypic and functional changes of pDCs after activation by IMQ and CpG, highly purified pDCs were generated from bone marrow of mice receiving a single injection of plasmid DNA encoding Flt3L, as previously described [28, 29]. First, the optimal concentration of IMQ and CpG was established to activate pDCs. Results revealed that 1.5 μM of IMQ and CpG in the optimal induction of MHC II, CD40, CD80, and CD86 (Supplementary Figure 1). Hence, we used the 1.5 uM concentration of IMQ and CpG in our subsequent studies. We used Giemsa staining to determine the morphological changes of pDCs after activation with IMQ and CpG (Figure 1A), and flow cytometry was used to detect phenotypic changes associated with activation of pDCs (Figure 1B). ELISA and CBA were used to characterize the functional changes of pDCs upon activation (Figure 1C). We demonstrated that the size of pDCs increased significantly after activation by IMQ (Figure 1A), and the expression of MHC II, CD40, CD80, and CD86 on pDCs was induced (Figure 1B). In contrast, after activation by CpG, only few pDCs revealed a change in size (Figure 1A); and the expression of MHC II, CD40, CD80, and CD86 on pDCs increased less than after activation with IMQ (Figure 1B). Both IMQ and CpG induced the release of IFN-α, IL-12p70, TNF-α, and IL-6 from pDCs (Figure 1C).

### Cytotoxicity of activated pDCs to tumor cells *in vitro*

**Figure 2:**
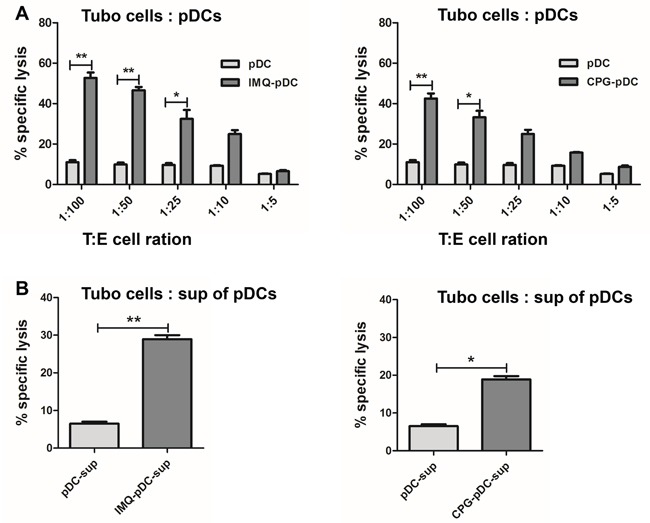
IMQ or CpG-activated pDCs kill tumor cells *in vitro* **A**. Sorted pDCs were stimulated by IMQ or CpG for 36 hours, and were co-cultured with TUBO cells for another 5 hours. Unstimulated pDCs were used as control. The killing activity was determined by cytotoxicity assay. **B**. Supernatant from activated pDCs were harvested after 36 hours, and added to TUBO cells for 5 hours. Killing activity was determined by cytotoxicity assay. Specific lysis means of 3 independent experiments performed in triplicates are represented ± SEM. Paired t-test was used for statistical comparison, *P<0.05, ^*^P<0.01.

Some studies have suggested that activated pDCs can inhibit melanoma growth [15]. In order to analyze whether pDCs are able to kill tumor cells after IMQ or CpG activation, pDCs were stimulated with IMQ or CpG, and co-cultured the pDCs with TUBO breast cancer cells. Either IMQ or CpG-stimulated pDCs showed increased cytotoxicity against TUBO cells, compared to unstimulated controls (Figure 2A); and the activation by IMQ resulted in stronger cytotoxicity than the activation by CpG. In order to determine whether the destruction of these cells was dependent on secreted cytotoxic molecules, TUBO cells were incubated with the supernatant of IMQ or CpG stimulated pDCs. Supernatant from activated pDCs were also able to kill TUBO cells, indicating that soluble, death inducing factors are produced by pDCs after activation with IMQ or CpG (Figure 2B). The cytotoxicity induced by IMQ was stronger than after activation with CpG.

### pDCs kill tumor cells in a TRAIL and Granzyme B-dependent fashion

**Figure 3:**
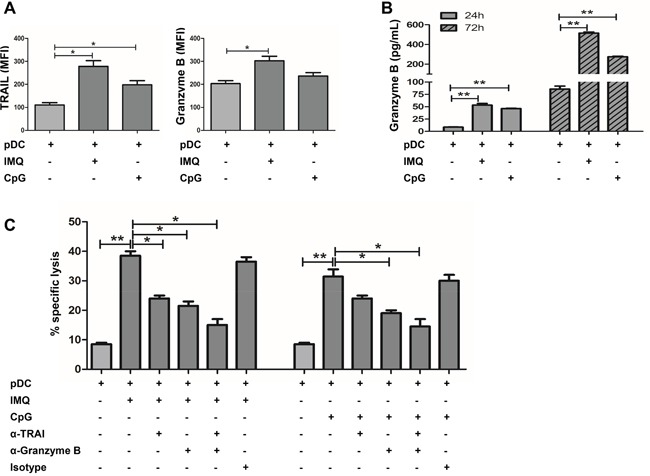
IMQ or CpG-activated pDCs kill TUBO cells in a TRAIL and Granzyme B-dependent fashion **A**. pDCs were stimulated by IMQ or CpG for 36 hours, and the expression of cytotoxic molecules TRAIL and Granzyme B on pDCs was assessed by flow cytometry and **B**. release of Granzyme B from pDCs by ELISA. Data shown are expressed as mean ± SEM. **C**. pDCs were stimulated by IMQ or CpG for 36 hours, and pre-incubated with neutralizing anti-TRAIL, anti-Granzyme B (10 ug/ml each) alone or combined, and isotype-matched control Abs for 30 minutes prior to the addition of TUBO cells. Cytotoxic activity was determined 5 hours later. Specific lysis means of 3 independent experiments performed in triplicates are represented ± SEM. Paired t-test was used for statistical comparison, *P<0.05, ^*^P<0.01.

Next, cytolytic molecules expressed by IMQ or CpG stimulated pDCs were determined. It is known that TLR7 and TLR9 ligands can stimulate the death-receptor ligand expression and secretion of biologically active TRAIL and Granzyme B by pDCs [15, 30]. Results in this study revealed that the IMQ or CpG stimulation of pDCs induced the expression of TRAIL, and that the effect of IMQ was stronger than that of CpG (Figure 3A). We also found that the IMQ or CpG stimulation of pDCs significantly induced the expression and release of Granzyme B, and that the effect of IMQ was also much stronger than that of CpG (Figure 3A and 3B). A significant decrease in the cytotoxic capacity of IMQ or CpG-stimulated pDCs was observed in the presence of the anti-Granzyme B antibody (Figure 3C). The antibody-mediated blockade of TRAIL also impaired the cytotoxicity of IMQ or CpG-activated pDCs. However, the effect of the TRAIL blockade was smaller than the blockade for Granzyme B (Figure 3C), especially in the CpG treated group. The cytotoxic capacity of IMQ or CpG-activated pDCs was significantly reduced by combination of anti-Granzyme B and anti-TRAIL antibodies. Although combined treatment was more potent than by a single antibody (Figure 3C), it did not completely block the cytotoxic capacity of activated pDCs. These data demonstrate that pDCs can kill tumor cells in a Granzyme B and TRAIL-dependent manner, however, other factors contribute to their cytotoxicity.

### Activated pDCs induce strong anti-tumor effects *in vivo*

**Figure 4:**
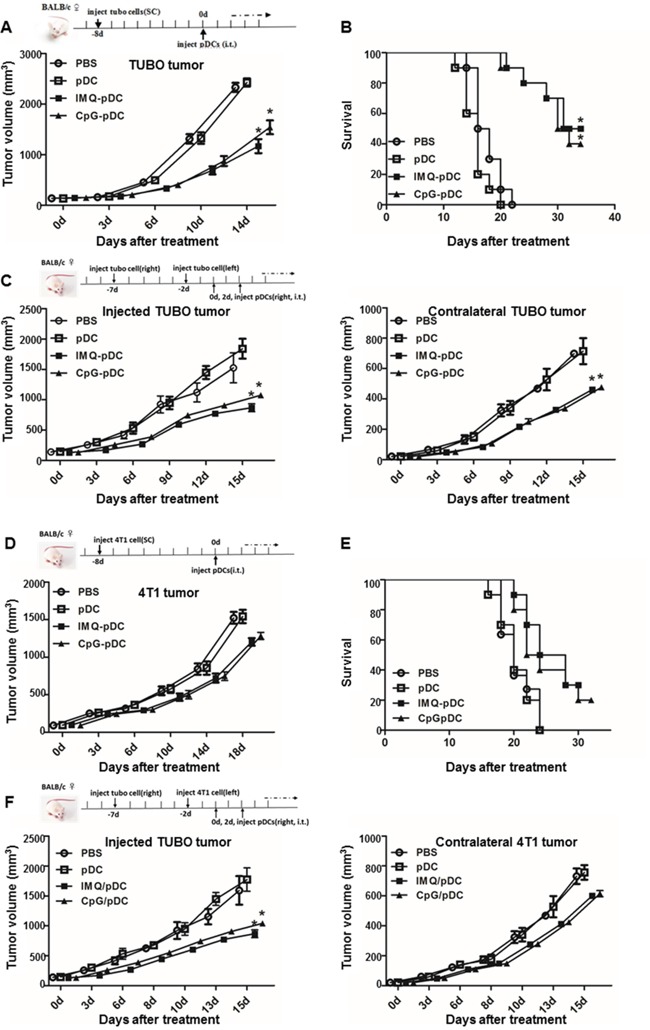
Administration of activated pDCs induces systemic anti-tumor activity TUBO or 4T1 bearing mice were treated by i.t. injection of IMQ or CpG activated pDCs, resting pDCs, or saline on day 0. **A, D**. Tumor growth and **B, E**. mice survival monitored over time is shown. **C, F**. Mice bearing subcutaneous TUBO or 4T1 tumors after injection of TUBO cells at day -7 into the right flank and at day -2 into the left flank were treated by i.t. injection of pDCs into the tumor on the right flank on day 0 and day 2. The graph depicts the growth of tumors on both flanks over time following treatment. Data show a representative of three independent experiments with similar results.

In order to determine whether pDCs are capable of triggering anti-tumor activity *in vivo*, the murine TUBO and 4T1 breast cancer cell lines were used [26]. BALB/c mice were inoculated subcutaneously with TUBO or 4T1 tumor cells, and established tumors were treated after 8 days with a single i.t. injection of IMQ or CpG-activated or resting pDCs, or saline (Figure 4A, 4D). Results revealed that the treatment of mice with activated pDCs, but not with resting pDCs or saline, led to a significant anti-tumor response (Figure 4A, 4D). The anti-tumor response of IMQ-activated pDCs was stronger than pDCs activated with CpG (Figure 4A). The survival of mice treated with pDCs was also significantly prolonged compared with the control group (Figure 4B, 4E). Again, mice treated with pDCs activated with IMQ survived longer than mice treated with pDCs activated with CpG. In order to determine whether the i.t. injection of pDCs was able to induce systemic anti-tumor activity, mice were subcutaneously inoculated with TUBO cells on their right flank on day -7, and inoculated TUBO or 4T1 cells on their left flank on day -2. Mice were treated with activated or resting pDCs or saline by i.t. injection into the tumors on their right flank on day 0 and 2 (Figure 4C, 4F). Results revealed that the tumor growth on both flanks was inhibited after the injection of activated pDCs, but not by resting pDCs or saline (Figure 4C, 4F). Anti-tumor response after treatment with IMQ-activated pDCs was stronger than after treatment with CpG-activated pDCs (Figure 4C).

### NK cell and CD8^+^ T cell involvement in the anti-tumor effect of pDCs *in vivo*

**Figure 5:**
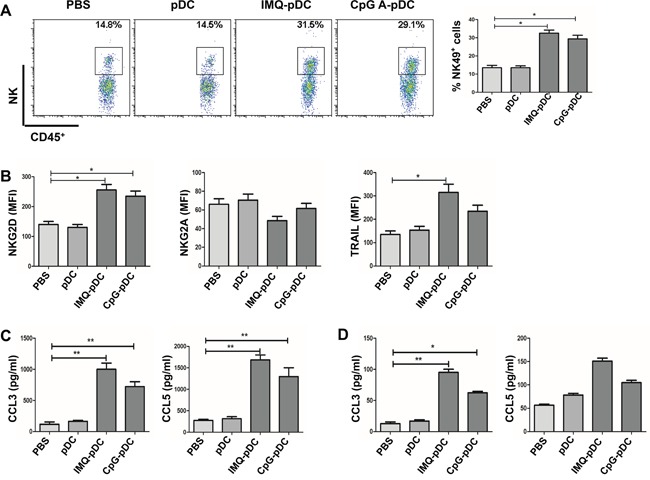
NK cells mediate the anti-tumor activity of pDCs **A**. Tumor-bearing mice were injected i.t. with IMQ or CpG-activated pDCs, resting pDCs, or with saline. Infiltration of NK cells at the tumor microenvironment was determined by flow cytometry on day 2. **B**. The expression of NK cell receptors NKG2D, NKG2A and cytotoxic molecule TRAIL were detected by flow cytometry on day 2. **C**. Chemotaxis of NK cells to tumor sites was induced by pDCs activated with IMQ or CpG for 36 hours. The levels of cytokine CCL3 and CCL5 in supernatant from pDCs were determined using CBA. **D**. Tumor-bearing mice were injected i.t. with IMQ or CpG-activated pDCs, resting pDCs, or saline. The level of cytokine CCL3 and CCL5 in serum of mice were determined using CBA. Data shown are expressed as mean ± SEM, and represent three independent experiments with similar results. Paired t-test was used for statistical comparison, *P<0.05, ^*^P<0.01.

**Figure 6:**
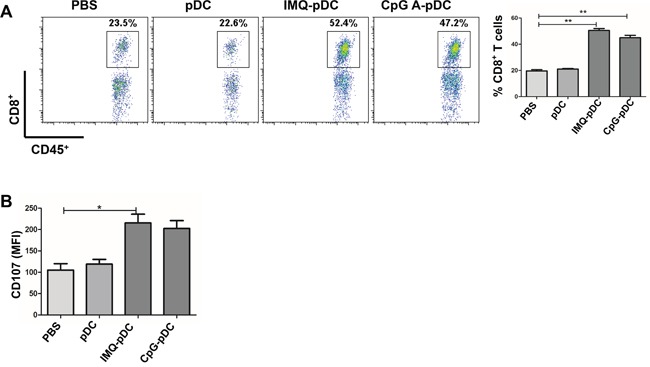
The role of CD8^+^ T cells in the anti-tumor activity of pDCs **A**. Tumor-bearing mice were injected with IMQ or CpG-activated pDCs, resting pDCs, or saline. The infiltration of CD8^+^ T cells at the tumor microenvironment were determined by flow cytometry on day 5. **B**. The expression of cytotoxic molecule CD107a on CD8^+^T cells was determined by flow cytometry on day 5. Data were shown as mean ± SEM. One of these three experiments is presented.

Next, the mechanism of the anti-tumor effect of activated pDCs *in vivo* was investigated. It was determined whether NK cells and CD8^+^ T cells play a role in the killing of tumor cells at early time points in the pDC treatment model in this study. The flow cytometric analysis of TUBO tumors following the administration of IMQ/CpG-activated pDCs revealed a dramatic infiltration of NK cells by day 2 (Figure 5A) and CD8^+^ T cells by day 5 (Figure 6A). In contrast, there was no difference in the number of CD8^+^ T cells by day 2 and NK cells by day 5 (data not shown). After the injection of activated pDCs, the number of infiltrating NK and CD8^+^ T cells also increased in the contralateral tumors, similar to the numbers in injected tumors (Supplementary Figure 2). Along with increased NK cell numbers, tumor-infiltrating NK cells also demonstrated an activated phenotype, as assessed by the expression of the activation molecule NKG2D and of the cytotoxic molecule TRAIL (Figure 5B), as well as the expression of the inhibitory molecule NKG2A (Figure 5B). Similarly, CD8^+^ T cells express the cytotoxic molecule CD107 (Figure 6B).

In order to further investigate the mechanism of the i.t. accumulation of NK cells, it was determined whether enhanced NK cell chemotaxis to tumor sites was induced by activated pDCs. The expression of chemokine CCL3 and CCL5 by activated pDCs were investigated, which all bind to the chemokine receptor CCR5 and is expressed on NK cells [14]. Furthermore, substantial amounts of chemokine CCL3 and CCL5 were detected in supernatant obtained from IMQ or CpG-activated pDCs and in serum from mice treated with activated pDCs (Figure 5C and 5D). These data demonstrate that activated pDCs may be involved in inducing NK cell chemotaxis to tumor sites.

## DISCUSSION

pDCs are major inducers of immune responses against viruses and bacteria through TLR7/9 activation. pDCs are capable of linking the innate and adaptive immune system *via* the rapid and sustained production of cytokines, and can activate T cells through direct antigen presentation. The present study revealed that after activation by IMQ and CpG, the expression of MHC II, CD40, CD80, and CD86 on pDCs significantly increased (Figure 1B). Accordingly, activated pDCs released increased amount of cytokines such as IFN-α, TNF-α, and IL-12p70 (Figure 1C). In addition, we found that after IMQ activation, the size of pDCs became dramatically enlarged, while no such change was observed after CpG activation (Figure 1A). The significant enlargement of pDCs may be indicative of their activation, associated with the upregulation of cytokine production and enhanced antigen presentation.

The role of pDCs in tumors has long been under debate. Clinical studies have shown an inverse correlation between the number of infiltrating pDCs and patient prognosis [13]. This was attributed to the finding that immature pDCs in tumors are weak inducers of T cell immunity or may even induce regulatory T cells [12, 31]. Furthermore, these cells are present in their non-activated state, and have been associated with the development and maintenance of an immunosuppressive tumor microenvironment. However, increasing evidence indicates that pDCs may play a critical role in tumor immunity.

It was reported that activated pDCs are capable of induce systemic anti-tumor activity through the activation of NK cells, cDCs and CD8^+^ T cells in the B16 mouse melanoma tumor model [14, 17]. In addition, activated pDCs can transform into a subset of killer DCs that are able to directly eliminate tumor cells in a mouse melanoma tumor model and in melanoma patients [15, 16]. Furthermore, we found that activated pDCs inhibited the local growth of TUBO or 4T1 (Figure 4A, 4D), and also inhibited the growth of contralateral inoculated TUBO or 4T1 tumors (Figure 4C, 4F). Activated pDCs directly destroyed breast tumor cells *in vitro* in a TRAIL and Granzyme B-dependent fashion. We demonstrated a robust infiltration of NK cells and CD8^+^ T cells in the TUBO tumor microenvironment (Figure 5A, Figure 6A), even in the contralateral tumor (Supplementary Figure 2). Thus, our data revealed that activated pDCs prevent breast cancer tumor growth in different models. We showed that activated pDCs not only kill local breast tumor directly, but also similar to mAbs induced systemic immune response [32] kill tumor contralaterally.

Several studies have demonstrated that IFNs are able to induce apoptosis in malignant cells, either by direct cytotoxic effects or by enhancing the expression of death-inducing molecules such as TRAIL, FasL and Granzyme B [33–35]. We found that IMQ-stimulated pDCs induced the expression of TRAIL and Granzyme B, and the release of Granzyme B; while CpG had a similar effect, but to a lesser extent (Figures 3A and 3B). A significant decrease in the cytotoxic capacity of IMQ or CpG-stimulated pDCs was observed in the presence of an anti-Granzyme B antibody (Figure 3C). The antibody-mediated blockade of TRAIL also impaired tumor cell killing by IMQ or CpG-induced pDCs. However, this was less efficient than the antibody blockade of Granzyme B (Figure 3C), especially in the CpG treated group. The cytotoxic capacity of IMQ or CpG-stimulated pDCs in the presence of both anti-Granzyme B and anti-TRAIL antibodies was much more inhibited than by a single antibody (Figure 3C), but they still did not totally block the cytotoxic capacity of IMQ or CpG-stimulated pDCs. This suggests that there are additional mechanisms involved in the cytotoxic activity of activated pDCs such as IFN-α, FasL, perforin, granulysin, and lysozyme.

Loschko *et al*. [35] have shown that the recognition of antigens by Siglec-H dampens pDC-induced adaptive responses. Siglec-H was described as a specific marker for pDCs, and an expression that can discriminate between proinflammatory and tolerogenic pDCs [35]. In our study, Siglec-H expression on pDCs significantly decreased after activation with IMQ or CpG, with the effect of IMQ being much stronger than the effect of CpG (Supplementary Figure 3). In agreement with this finding, the lower expression of Siglec-H on activated pDCs correlated with the lower presence of immunosuppressive cells and cytokines that facilitate tumor development [35].

At present, there is no published research that compared the anti-tumor effects of pDCs activated by either the TLR7 ligand IMQ or the TLR9 ligand CpG. In the present study, IMQ-activated pDCs elicited stronger anti-tumor responses in mice than CpG-activated pDCs. This may be related to the cytolytic molecules Granzyme B and TRAIL. We observed that IMQ induced the expression and release of Granzyme B and TRAIL in pDCs much more potently than CpG (Figures 3A and 3B). Furthermore, it has been reported that IMQ induces the expression of Granzyme B and TRAIL on pDCs sufficiently to effectively lyse appropriate tumor targets [36]. In the future, further studies to determine whether combined IMQ and CpG treatment can have an even stronger anti-tumor function are warranted.

Stary G e*t al* [36] reported that TRAIL and Granzyme B secretion by pDCs is induced *via* IFNAR1 signaling. Drobits *et al* [15] reported that pDCs-mediated killing of melanoma requires IFNAR1 signaling. We found that IMQ and CpG induced the release of IFN-α from pDCs (Figure 1C). In the future, we will further explore whether type-I IFNs have the potential to unleash anti-tumor immunity by activating conventional DCs, T cells and NK cells, while inhibiting regulatory T cells, and whether type-I IFNs can induce the cytotoxic activities of pDCs by inducing the expression of Granzyme B and TRAIL, leading to local anti-tumor responses in the breast tumor mouse model in this study and in breast cancer patients.

In conclusion, our data provides strong evidence that pDCs are required for the anti-tumor response mediated by IMQ and CpG. Activated pDCs can directly kill breast tumor cells in a TRAIL and Granzyme B-dependent fashion. Furthermore, pDCs activate NK cells and CD8^+^ T cells, and ultimately inhibit breast tumor growth. Understanding the function of activated pDCs in anti-tumor immunity in breast cancer may shed new light on the development of a new strategy to manipulate the function of pDCs and induce anti-tumor immunity in breast cancer.

## MATERIALS AND METHODS

### Mice and cell lines

Female BALB/c mice were purchased from Beijing WeiTongLiHua Laboratory and maintained in a pathogen-free animal facility at the Institute of Translational Medicine, The First Hospital, Jilin University. The mice used in this study were 6-8 weeks of age, and all animal experiments were performed according to protocols approved by the Institutional Animal Care and Use Committee of the University of Jilin. The TUBO tumor cell line was cloned from a spontaneous mammary tumor in a BALB HER2/Neu Tg mouse. Another spontaneous mammary tumor model 4T1 was used. The cell lines were a gift from Dr. Liguo Zhang (Chinese Academy of Sciences, Beijing). TUBO and 4T1 cells were cultured in DMEM supplemented with 10% heat-inactivated FBS, 1% sodium pyruvate, 1% nonessential amino acids, and 1% penicillin-streptomycin (Invitrogen, USA).

### Isolation and culture of pDCs

Murine pDCs were isolated from the bone marrow of Flt3L-treated mice. An expression vector encoding full-length murine Flt3L cDNA (pORF-mFlt3L) was purchased from (Invitrogen, USA). 10μg of plasmid DNA encoding Flt3L was injected using the hydrodynamic-based gene delivery technique, as previously described [28]. Bone marrow cells were isolated from the femora and tibiae 10 days after Flt3L plasmid injection, incubated with rat anti-CD16/32 mAbs to block nonspecific binding, and stained with the following mAbs: anti-CD11c, anti-B220, and anti-CD11b (BD Biosciences, USA). pDCs with a CD11c^int^CD11b^–^B220^+^ phenotype were sorted using a BD FACSAria. The purity of the isolated pDC population was generally >99%. Cell viability determined by trypan blue staining was >99% after isolation. pDCs were cultured at 2.5×10^6^ cells/mL in RPMI 1640 medium supplemented with 10% FBS, 1% Pen/Strep, 1% nonessential amino acids, 1% sodium pyruvate, and 0.1% β-mercaptoethanol. pDCs were harvested after activation with IMQ and CpG for 48 hours; and were assessed for morphologic changes after Giemsa staining and for phenotypic changes by flow cytometry.

### Murine mammary tumor model

BALB/c mice (5 mice per group) were subcutaneously inoculated with 5×10^5^ TUBO tumor cells or 2×10^5^ 4T1 tumor cells on day -8 (Figure 4A, 4D). Tumor-bearing mice were treated on day 0 by intratumoral (i.t.) injection of 2×10^6^ resting pDCs or with pDCs activated for 5 hours with IMQ or CpG, which were washed with PBS before the *in vivo* transfer. Tumor growth and mouse survival were monitored. In order to investigate whether i.t. injection of pDCs could induce systemic anti-tumor activity, mice were subcutaneously inoculated with 4×10^5^ TUBO cells on their right flank on day -7 and 2×10^5^ TUBO or 1×10^5^ 4T1 tumor cells on their left flank on day -2 (Figure 4C, 4F). Mice were treated with 2×10^6^ resting pDCs or with pDCs activated for 5 hours with IMQ or CpG by i.t. injection in tumors on the right flank on day 0 and 2. Tumor volumes were measured along three orthogonal axes (a, b and c), and were calculated as tumor volume = abc/2. Mice were sacrificed when tumors reached volumes of more than 2,000 mm^3^.

### Flow cytometry analysis

pDCs were harvested after activation with IMQ and CpG for 48 hours, and stained with the following mAbs: anti-CD11c, anti-CD11b, anti-B220, anti-CD80, anti-CD86, anti-MHC II (BD Pharmingen, USA), anti-TRAIL, and anti-Granzyme B (eBioscience, USA). Mice were injected i.t. with resting or activated pDCs, and sacrificed on day 2 or 5. Single-cell suspensions were prepared from tumor tissues. After enzymatic digestion for 30 minutes at 37°C with type IA collagenase (1 mg/mL) and DNase (0.1 mg/mL), red blood cells were lysed with Pharmlyse Buffer (BD Biosciences, USA); and stained with the following mAbs: anti-CD45, anti-NK, anti-CD3, anti-CD8, anti-TRAIL, anti-NKG2D, anti-NKG2A, and CD107 (BD Pharmingen, USA). Intracellular staining was performed using a cell permeabilization kit (Fix & Perm; An Der Grub). After incubation with the respective Abs for 20 minutes at 4°C, cells were washed twice and subjected to flow cytometric analysis. FACS plots depict the mean fluorescence intensity (MFI) values of Ab staining after subtraction of the MFI of the respective isotype.

### ELISA and CBA assay

pDCs were cultured at 2.5×10^6^ cells/mL with IMQ and CpG in RPMI medium supplemented with 10% FBS, 1% Pen/Strep, 1% nonessential amino acids, 1% sodium pyruvate, and 0.1% β-mercaptoethanol. Supernatant were collected after 36 hours, and analyzed with the following ELISA kits: mouse IFN-α (R&D Systems, USA), and mouse Granzyme B (Elabscience, USA). The levels of cytokine IL-12p70, TNF-α, and IL-6, as well as the levels of chemokine CCL3 and CCL5, in supernatant from resting or activated pDCs were analyzed using a cytometric bead array (CBA; BD, USA). The levels of chemokine CCL3 and CCL5 in serum from mice treated with resting or activated pDCs were analyzed using CBA. Briefly, 50 μl of samples or recombinant standards were added to 50 μl of mixed capture beads, and incubated for 3 hours with 50 μl of phycoerythrin-conjugated detection antibodies (Ab-PE) to form sandwich complexes. After washing to remove unbound Ab-PE detection reagent and performing FACS analysis using FACSArray (BD, USA), results were generated using FCAP Array software (BD, USA).

### Cytotoxicity assays

The ability of pDCs to destroy tumor cells was assessed in a classic Europium-TDA release assay (DELFIA; PerkinElmer), according to manufacturer's instructions. Briefly, target cells were labeled with the fluorescence-enhancing ligand BATDA, which was released into the supernatant after cytolysis. The supernatant was harvested and incubated with the Europium solution to form a stable fluorescent chelate. Data were obtained using the 1234 DELFIA Luminometer (Wallac), and are expressed as the percentage of specific lysis calculated by the following formula: specific lysis (%) = (experimental release - spontaneous release) / (maximum release - spontaneous release) × 100. TUBO cells were used as target cells and pDCs were used as effector cells. Purified pDCs that were been stimulated with IMQ and CpG for 36 hours, or had been left unstimulated were incubated with 5×10^3^ target cells in duplicate in 96-well plates at E: T ratios ranging from 100:1 to 5:1. Target cell lysis was measured after 5 hours. In order to evaluate the contribution of soluble cytolytic molecules present in the supernatant of pDCs cultures to pDCs killer activity, cytotoxicity assays were performed using the supernatant of IMQ/CpG activated pDCs (cultured at 1×10^6^ cells/ml) that were added to 2×10^3^ target cells. Inhibition experiments were performed by preincubating pDCs with neutralizing anti-Granzyme B (10 ug/ml), or anti-TRAIL (10 ug/ml) antibodies alone or combined, or with an isotype-matched non-specific Ab for 30 minutes prior to the addition of target cells.

### Statistical analysis

Data are expressed as mean ± SD or SEM, as indicated. Differences between experimental groups were analyzed using two-tailed Student *t*-test, and *P*-values <0.05 were considered significant (*P<0.05, ^*^P<0.01, ^**^*P<0.001).

## SUPPLEMENTARY MATERIALS FIGURES



## Abbreviations

DCs: Dendritic cells
mDCs: myeloid dendritic cells
pDCs: plasmacytoid dendritic cells
NK: natural killer cells
IMQ: Imiquimod
MFI: mean fluorescence intensity
CBA: cytometric bead array analysis
TRAIL: TNF-related apoptosis-inducing ligand
