# Supplementary material for: TLR-activated plasmacytoid dendritic cells inhibit breast cancer cell growth in vitro and in vivo
Source: Oncotarget. 2016 Dec 28;8(7):11708–18. doi: 10.18632/oncotarget.14315 (PMC5355297; doi:10.18632/oncotarget.14315)
Supplement: Supplementary file 1 [file oncotarget-08-11708-s001.pdf]

TLR-activated plasmacytoid dendritic cells inhibit breast cancer cell growth *in vitro* and *in vivo*

SUPPLEMENTARY FIGURES

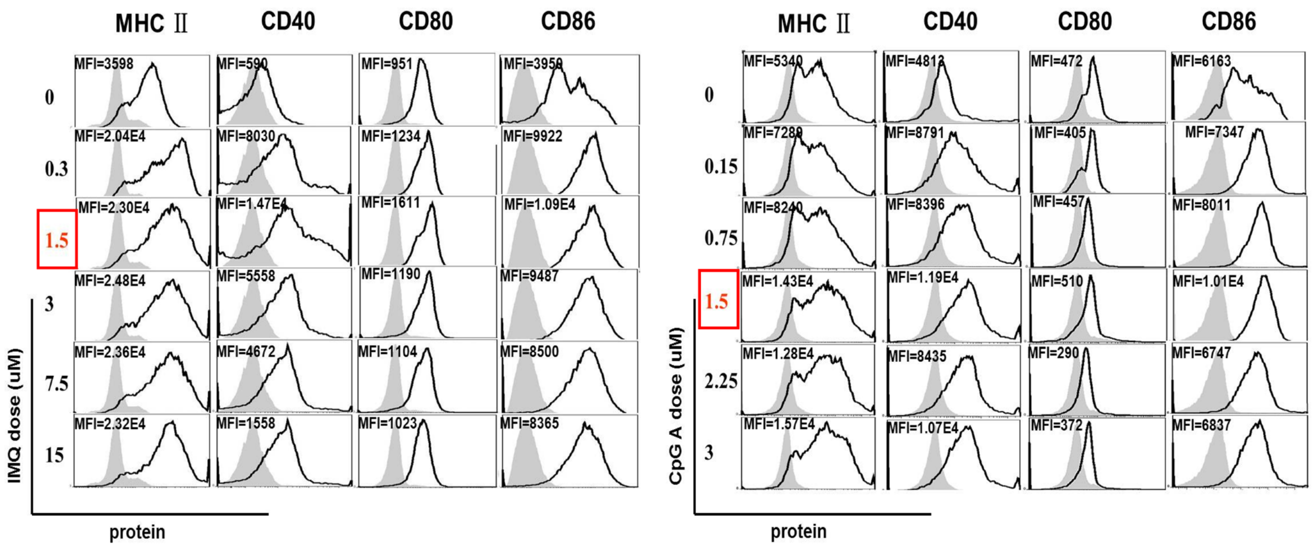

Supplementary Figure 1: pDCs phenotypic change after activation by different doses of IMQ (0, 0.3, 1.5, 3, 7.5 and 15 μM) and CpG (0, 0.15, 0.75, 1.5, 2.25 and 3 μM). pDCs were harvested after activation with IMQ **A.** and CpG **B.** for 48 hours, and were assessed for phenotypic changes by flow cytometry. Data show a representative of three independent experiments with similar results.

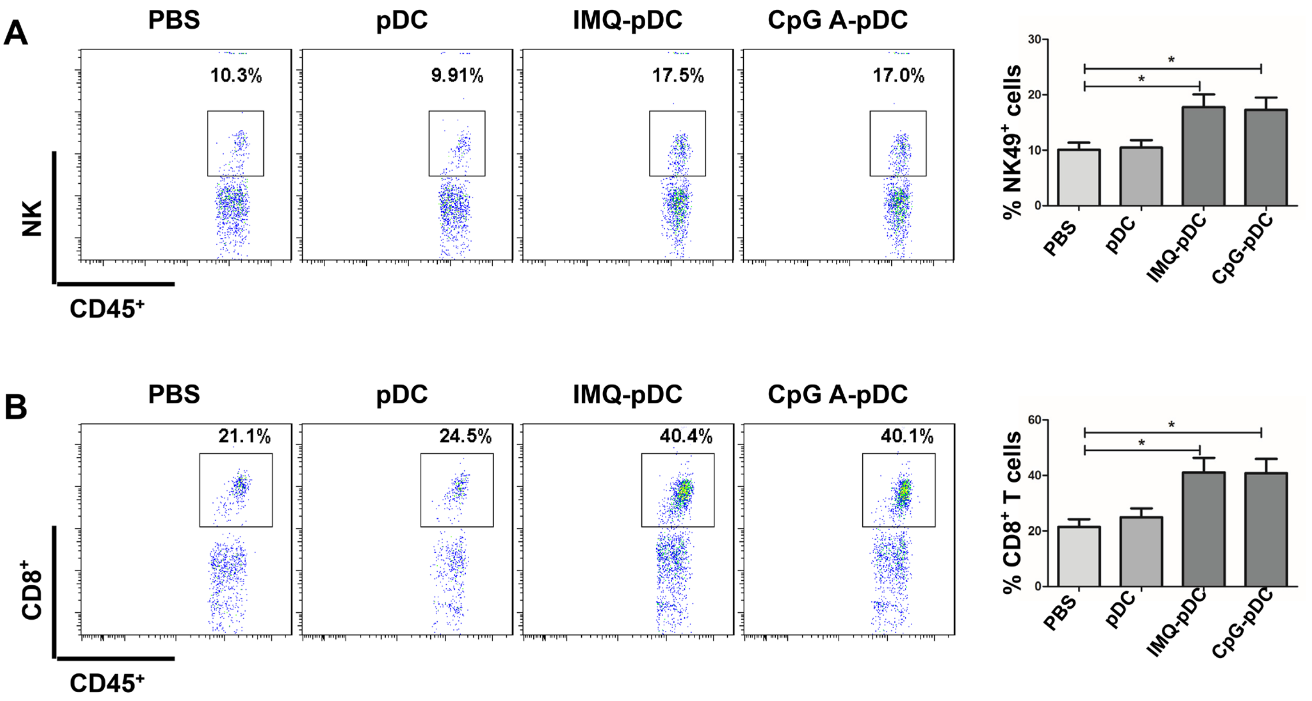

Supplementary Figure 2: NK and CD8+ T cells mediate the anti-tumor activity of pDCs. Mice were subcutaneously inoculated with 4×10^5 TUBO cells on their right flank on day-7 and 2×10^5 TUBO on their left flank on day-2. Mice were treated with 2×10^6 resting pDCs or with pDCs activated for 5 hours with IMQ or CpG by i.t. injection in tumors on the right flank on days 0 and 2. Infiltration of NK and CD8+ T cells at the contralateral tumor microenvironment was determined by flow cytometry. One of these three experiments is presented.

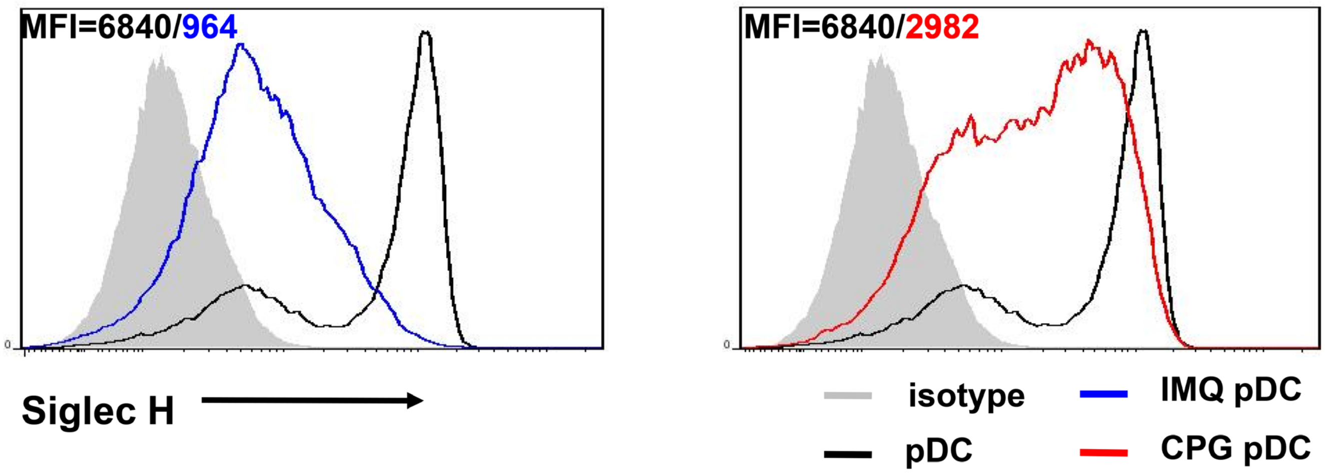

Supplementary Figure 3: The expression of Siglec H on pDCs after activation with IMQ and CpG. pDCs were harvested after activation with IMQ and CpG for 48 hours, and assessed the expression of Siglec H on pDCs by flow cytometry. One of these three experiments is presented.
